# Supplementary material for: Metastasis, characteristic, and treatment of breast cancer in young women and older women: A study from the Surveillance, Epidemiology, and End Results registration database
Source: PLoS One. 2023 Nov 2;18(11):e0293830. doi: 10.1371/journal.pone.0293830 (PMC10621871; doi:10.1371/journal.pone.0293830)
Supplement: S1 Table — (DOCX) [file pone.0293830.s001.docx]

**Table S1. Covariates related to OS and CSS in young patients with BC**

| Variables | OS | | CSS | |
| --- | --- | --- | --- | --- |
|  | HR^#^ (95% CI) | *P* | HR^#^ (95% CI) | *P* |
| Age | 0.99 (0.97-1.01) | 0.375 | 0.99 (0.97-1.01) | 0.559 |
| Race |  |  |  |  |
| White | Ref |  | Ref |  |
| Black | 1.65 (1.33-2.03) | <0.001 | 1.50 (1.20-1.88) | <0.001 |
| Other/unknown | 0.92 (0.69-1.23) | 0.568 | 0.93 (0.70-1.24) | 0.627 |
| Marital status |  |  |  |  |
| Married (including common law) | Ref |  | Ref |  |
| Unmarried (including separated, divorced, widowed, single) | 1.25 (1.04-1.50) | 0.016 | 1.22 (1.01-1.46) | 0.036 |
| Unknown | 1.64 (1.10-2.45) | 0.014 | 1.72 (1.18-2.51) | 0.005 |
| Family income |  |  |  |  |
| < $70000 | Ref |  | Ref |  |
| ≥ $70000 | 0.73 (0.61-0.89) | 0.001 | 0.77 (0.64-0.94) | 0.008 |
| Primary site |  |  |  |  |
| Central portion | Ref |  | Ref |  |
| LIQ | 1.04 (0.57-1.91) | 0.887 | 1.05 (0.56-1.97) | 0.887 |
| LOQ | 1.21 (0.70-2.09) | 0.503 | 1.15 (0.66-2.00) | 0.618 |
| UIQ | 0.70 (0.38-1.29) | 0.254 | 0.66 (0.35-1.26) | 0.205 |
| UOQ | 0.97 (0.59-1.57) | 0.887 | 0.93 (0.56-1.54) | 0.770 |
| Unknown | 1.11 (0.70-1.76) | 0.666 | 1.03 (0.63-1.68) | 0.911 |
| Tumor size |  |  |  |  |
| ≤ 2 | Ref |  | Ref |  |
| 2-5 | 1.61 (1.10-2.35) | 0.014 | 1.65 (1.14-2.40) | 0.009 |
| > 5 | 2.39 (1.63-3.52) | <0.001 | 2.29 (1.56-3.36) | <0.001 |
| Unknown | 1.75 (1.19-2.58) | 0.004 | 1.62 (1.11-2.36) | 0.012 |
| Tumor grade |  |  |  |  |
| I | Ref |  | Ref |  |
| II | 0.85 (0.48-1.51) | 0.590 | 0.80 (0.47-1.36) | 0.406 |
| III | 1.30 (0.74-2.26) | 0.361 | 1.26 (0.75-2.11) | 0.387 |
| IV | 1.45 (0.60-3.49) | 0.412 | 1.46 (0.52-4.06) | 0.471 |
| Unknown | 1.45 (0.81-2.58) | 0.214 | 1.27 (0.74-2.18) | 0.387 |
| AJCC T stage |  |  |  |  |
| T1 | Ref |  | Ref |  |
| T2 | 1.72 (1.12-2.66) | 0.014 | 1.79 (1.18-2.71) | 0.007 |
| T3 | 2.09 (1.32-3.29) | 0.002 | 2.02 (1.29-3.15) | 0.002 |
| T4 | 2.63 (1.68-4.11) | <0.001 | 2.60 (1.68-4.03) | <0.001 |
| TX | 2.81 (1.69-4.66) | <0.001 | 2.93 (1.80-4.78) | <0.001 |
| Unknown | 1.85 (1.18-2.90) | 0.008 | 1.67 (1.08-2.57) | 0.021 |
| AJCC N stage |  |  |  |  |
| N0 | Ref |  | Ref |  |
| N1 | 0.91 (0.69-1.20) | 0.499 | 0.90 (0.68-1.19) | 0.447 |
| N2 | 0.68 (0.47-0.97) | 0.034 | 0.69 (0.48-0.99) | 0.044 |
| N3 | 1.16 (0.84-1.60) | 0.358 | 1.09 (0.78-1.52) | 0.604 |
| NX | 0.85 (0.51-1.43) | 0.544 | 0.80 (0.47-1.36) | 0.416 |
| Unknown | 0.85 (0.63-1.16) | 0.309 | 0.75 (0.55-1.03) | 0.072 |
| Histological type |  |  |  |  |
| IDC | Ref |  | Ref |  |
| ILC | 0.80 (0.48-1.31) | 0.372 | 0.85 (0.52-1.39) | 0.513 |
| IDLC | 0.76 (0.39-1.47) | 0.420 | 0.81 (0.42-1.56) | 0.531 |
| Others | 1.42 (1.10-1.83) | 0.006 | 1.44 (1.11-1.88) | 0.007 |
| Subtype |  |  |  |  |
| HR^*^+/HER2+ | Ref |  | Ref |  |
| HR^*^+/HER2- | 2.21 (1.70-2.86) | <0.001 | 2.25 (1.74-2.90) | <0.001 |
| HR^*^-/HER2+ | 1.44 (1.01-2.07) | 0.045 | 1.45 (1.01-2.09) | 0.045 |
| HR^*^-/HER2- | 6.99 (5.15-9.48) | <0.001 | 6.24 (4.43-8.80) | <0.001 |
| Unknown | 2.54 (1.72-3.76) | <0.001 | 2.57 (1.71-3.86) | <0.001 |
| Metastatic site |  |  |  |  |
| Bone only | Ref |  | Ref |  |
| Lung only | 3.66 (2.04-6.57) | <0.001 | 3.73 (1.74-8.00) | <0.001 |
| Liver only | 0.74 (0.33-1.68) | 0.475 | 0.75 (0.33-1.71) | 0.493 |
| Brain only | 0.80 (0.58-1.12) | 0.193 | 0.80 (0.57-1.11) | 0.174 |
| Other distant sites | 1.51 (1.09-2.11) | 0.014 | 1.44 (1.01-2.04) | 0.042 |
| Multiple sites | 2.03 (1.67-2.48) | <0.001 | 1.84 (1.51-2.25) | <0.001 |
| Chemotherapy |  |  |  |  |
| Yes | Ref |  | Ref |  |
| No/unknown | 1.68 (1.35-2.10) | <0.001 | 1.65 (1.33-2.06) | <0.001 |
| Surgery |  |  |  |  |
| BCS | Ref |  | Ref |  |
| No | 0.70 (0.53-0.92) | 0.012 | 0.68 (0.51-0.91) | 0.009 |
| Mastectomy | 1.94 (1.60-2.35) | <0.001 | 1.86 (1.54-2.26) | <0.001 |
| Unknown type | 2.25 (1.23-4.13) | 0.009 | 2.35 (1.16-4.77) | 0.018 |
| Radiotherapy |  |  |  |  |
| Yes | Ref |  | Ref |  |
| No | 1.01 (0.84-1.22) | 0.899 | 0.98 (0.81-1.20) | 0.875 |

OS: overall survival, CSS: cancer-specific survival, BC: breast cancer, HR^#^: hazard ratio, CI: confidence interval, Ref: reference, LIQ: lower inner quadrant, LOQ: lower outer quadrant, UIQ: upper inner quadrant, UOQ: upper outer quadrant, AJCC: the American Joint Committee on Cancer stage, IDC: infiltrating ductal carcinoma, ILC: infiltrating lobular carcinoma, IDLC: infiltrating ductal mixed lobular carcinoma, HR^*^: hormonal receptor, HER: hormonal estrogen receptor, BCS: breast-conserving surgery.
