# Supplementary material for: Metastasis, characteristic, and treatment of breast cancer in young women and older women: A study from the Surveillance, Epidemiology, and End Results registration database
Source: PLoS One. 2023 Nov 2;18(11):e0293830. doi: 10.1371/journal.pone.0293830 (PMC10621871; doi:10.1371/journal.pone.0293830)
Supplement: S2 Table — (DOCX) [file pone.0293830.s002.docx]

**Table S2. Covariates related to OS and CSS in older patients with BC**

| Variables | OS | | CSS | |
| --- | --- | --- | --- | --- |
|  | HR^#^ (95% CI) | *P* | HR^#^ (95% CI) | *P* |
| Age | 1.02 (1.02-1.02) | <0.001 | 1.02 (1.01-1.02) | <0.001 |
| Race |  |  |  |  |
| White | Ref |  | Ref |  |
| Black | 1.30 (1.21-1.40) | <0.001 | 1.27 (1.18-1.37) | <0.001 |
| Other/unknown | 0.82 (0.74-0.91) | <0.001 | 0.84 (0.75-0.93) | <0.001 |
| Marriage |  |  |  |  |
| Married (including common law) | Ref |  | Ref |  |
| Unmarried (including separated, divorced, widowed, single) | 1.40 (1.33-1.48) | <0.001 | 1.32 (1.25-1.40) | <0.001 |
| Unknown | 1.19 (1.05-1.35) | 0.007 | 1.12 (0.98-1.28) | 0.102 |
| Family income |  |  |  |  |
| < $70000 | Ref |  | Ref |  |
| ≥ $70000 | 0.86 (0.81-0.91) | <0.001 | 0.86 (0.82-0.92) | <0.001 |
| Primary site |  |  |  |  |
| Central portion | Ref |  | Ref |  |
| LIQ | 0.87 (0.73-1.04) | 0.134 | 0.84 (0.71-1.01) | 0.059 |
| LOQ | 0.81 (0.69-0.95) | 0.010 | 0.82 (0.70-0.96) | 0.012 |
| UIQ | 0.89 (0.77-1.04) | 0.148 | 0.90 (0.77-1.05) | 0.174 |
| UOQ | 0.98 (0.87-1.10) | 0.698 | 0.97 (0.87-1.09) | 0.616 |
| Unknown | 1.15 (1.03-1.28) | 0.013 | 1.14 (1.02-1.26) | 0.016 |
| Tumor size |  |  |  |  |
| ≤ 2 | Ref |  | Ref |  |
| 2-5 | 1.05 (0.95-1.16) | 0.368 | 1.04 (0.93-1.16) | 0.466 |
| > 5 | 1.36 (1.22-1.51) | <0.001 | 1.38 (1.23-1.54) | <0.001 |
| Unknown | 1.24 (1.12-1.37) | <0.001 | 1.19 (1.07-1.33) | 0.001 |
| Tumor grade |  |  |  |  |
| I | Ref |  | Ref |  |
| II | 1.16 (1.02-1.32) | 0.023 | 1.29 (1.13-1.46) | <0.001 |
| III | 1.75 (1.54-1.98) | <0.001 | 1.94 (1.71-2.21) | <0.001 |
| IV | 2.08 (1.49-2.91) | <0.001 | 2.40 (1.65-3.49) | <0.001 |
| Unknown | 1.66 (1.46-1.89) | <0.001 | 1.76 (1.54-2.01) | <0.001 |
| AJCC T stage |  |  |  |  |
| T1 | Ref |  | Ref |  |
| T2 | 1.04 (0.92-1.17) | 0.510 | 1.02 (0.90-1.15) | 0.771 |
| T3 | 1.24 (1.09-1.41) | <0.001 | 1.25 (1.10-1.43) | <0.001 |
| T4 | 1.63 (1.46-1.83) | <0.001 | 1.59 (1.41-1.79) | <0.001 |
| TX | 1.61 (1.41-1.85) | <0.001 | 1.60 (1.38-1.85) | <0.001 |
| Unknown | 1.19 (1.06-1.34) | 0.003 | 1.12 (0.99-1.26) | 0.075 |
| AJCC N stage |  |  |  |  |
| N0 | Ref |  | Ref |  |
| N1 | 0.98 (0.90-1.06) | 0.618 | 1.00 (0.92-1.09) | 0.968 |
| N2 | 0.91 (0.82-1.01) | 0.070 | 0.90 (0.81-1.00) | 0.051 |
| N3 | 1.00 (0.91-1.11) | 0.958 | 1.02 (0.92-1.13) | 0.720 |
| NX | 1.40 (1.23-1.59) | <0.001 | 1.34 (1.17-1.54) | <0.001 |
| Unknown | 0.92 (0.84-1.00) | 0.060 | 0.88 (0.80-0.97) | 0.007 |
| Histological type |  |  |  |  |
| IDC | Ref |  | Ref |  |
| ILC | 0.87 (0.76-0.99) | 0.035 | 0.90 (0.79-1.03) | 0.122 |
| IDLC | 0.99 (0.90-1.09) | 0.830 | 0.99 (0.91-1.09) | 0.871 |
| Other | 1.44 (1.34-1.54) | <0.001 | 1.39 (1.29-1.49) | <0.001 |
| Subtype |  |  |  |  |
| HR^*^+/HER2+ | Ref |  | Ref |  |
| HR^*^+/HER2- | 1.30 (1.20-1.42) | <0.001 | 1.25 (1.15-1.37) | <0.001 |
| HR^*^-/HER2+ | 1.31 (1.15-1.48) | <0.001 | 1.27 (1.11-1.44) | <0.001 |
| HR^*^-/HER2- | 3.09 (2.79-3.42) | <0.001 | 2.93 (2.63-3.26) | <0.001 |
| Unknown | 2.42 (2.17-2.70) | <0.001 | 2.28 (2.02-2.56) | <0.001 |
| Metastatic site |  |  |  |  |
| Bone only | Ref |  | Ref |  |
| Lung only | 2.22 (1.88-2.62) | <0.001 | 2.29 (1.89-2.77) | <0.001 |
| Liver only | 0.83 (0.67-1.03) | 0.085 | 0.84 (0.68-1.03) | 0.100 |
| Brain only | 1.00 (0.88-1.14) | 0.971 | 1.01 (0.88-1.16) | 0.891 |
| Other distant sites | 1.29 (1.17-1.43) | <0.001 | 1.24 (1.12-1.38) | <0.001 |
| Multiple sites | 2.01 (1.90-2.13) | <0.001 | 2.02 (1.91-2.15) | <0.001 |
| Chemotherapy |  |  |  |  |
| Yes | Ref |  | Ref |  |
| No/unknown | 1.65 (1.56-1.74) | <0.001 | 1.51 (1.42-1.59) | <0.001 |
| Surgery |  |  |  |  |
| BCS | Ref |  | Ref |  |
| No | 0.92 (0.84-1.00) | 0.054 | 0.92 (0.84-1.00) | 0.054 |
| Mastectomy | 1.87 (1.76-1.99) | <0.001 | 1.82 (1.71-1.93) | <0.001 |
| Unknown type | 1.30 (1.03-1.63) | 0.026 | 1.29 (1.04-1.61) | 0.022 |
| Radiotherapy |  |  |  |  |
| Yes | Ref |  | Ref |  |
| No | 0.93 (0.88-0.98) | 0.010 | 0.89 (0.84-0.94) | <0.001 |

OS: overall survival, CSS: cancer-specific survival, BC: breast cancer, HR^#^: hazard ratio, CI: confidence interval, Ref: reference, LIQ: lower inner quadrant, LOQ: lower outer quadrant, UIQ: upper inner quadrant, UOQ: upper outer quadrant, AJCC: the American Joint Committee on Cancer stage, IDC: infiltrating ductal carcinoma, ILC: infiltrating lobular carcinoma, IDLC: infiltrating ductal mixed lobular carcinoma, HR^*^: hormonal receptor, HER: hormonal estrogen receptor, BCS: breast-conserving surgery.
