# Supplementary material for: Metastasis, characteristic, and treatment of breast cancer in young women and older women: A study from the Surveillance, Epidemiology, and End Results registration database
Source: PLoS One. 2023 Nov 2;18(11):e0293830. doi: 10.1371/journal.pone.0293830 (PMC10621871; doi:10.1371/journal.pone.0293830)
Supplement: Attachment — Submitted filename: Response to reviewers comments.docx [file pone.0293830.s003.docx]

**Responses to Reviewers**

Dear Editor and reviewers,

We highly appreciate and thank the Editors and the reviewers for your time and valuable comments on this manuscript. We have carefully studied the comments and suggestions and revised our paper accordingly. The following are our point-by-point responses to the comments. We hope that the revisions are acceptable and that our responses adequately address the comments. Thank you for your consideration.

**Responses to Reviewer #1’s comments**

The study assesses a current, timely topic in breast cancer.

We recommend some changes:

- We believe this article is suitable for publication in the journal although major revisions are needed. The main strengths of this paper are that it addresses an interesting and very timely question and provides a clear answer, with some limitations. Certainly, the authors should better highlight the limitations of the current paper. In addition, the samples are quite old.

Response: Thank you for your approbation and valuable comments. We have revised this manuscript to better highlight the limitations of the current study. The study sample were extracted from the SEER database, which includes the large numbers of representative population in the United States. SEER uses data collected and maintained by the National Center for Health Statistics (NCHS), which is from the Population Estimates Program (PEP) data of the United States Census Bureau and mortality data in the United States. SEER database routinely collects and publishes data on patient-specific and tumor-specific characteristics for about 30 percent of the US population. We are very sorry for the clerical error that the actual data collection time was 2010-2019. Due to the information of patients such as metastatic sites were collected from 2010, and the SEER database has not public data from 2020 and beyond, wo could only used the data from 2010 to 2019 when this study started.

- A linguistic revision is needed.

Response: Thank you for your comment. We have checked and revised the grammatical mistakes in the manuscript, and invited a native English speaker to help us. We hope the revised manuscript could meet your standards.

- The background of the changing scenario of medical treatment in breast cancer should be better discussed, and some recent papers regarding this topic should be included (PMID: 35171746; PMID: 36633661; PMID: 34802383; PMID: 34793275).

Major changes are necessary.

Response: Thank you for your valuable comment. We have read the references you provided, and added them into the discussion section.

**Responses to Reviewer #2’s comments**

Number 3. Datasets - It is concerning that, although the authors say all the datasets are available from SEER, the ones authors generated and/or analyzed are not SEER datasets, but their analysis. Are they available?

Response: Thank you for your comment. This was a retrospective cohort study, where the information of participants was extracted from the SEER database, which routinely collects and publishes data on patient-specific and tumor-specific characteristics for about 30 percent of population in the United States. The SEER database was publicly available, and the information of patients was all de-identified.

- The authors do not define "younger" and "older" patients by age until into the Introduction. This should be done in the Abstract. Also, OS and CSS are not defined, although one can presume, they mean Overall Survival and Cancer Specific Survival. In the Results section, it is stated that IDC was the most common histological type; one assumes the authors mean Invasive Ductal Carcinoma, which also should be spelled out.

Response: Thank you for your valuable comments. We have revised the abstract section and added the definition of “younger” and “older” patients by age as well as that of OS and CSS. We have revised the “infiltrating ductal carcinoma” into “invasive ductal carcinoma”.

Number 4 - There are problems with the language used by the authors. It is usually understandable in the sense that one can figure out what the authors mean, but they need to have a native English speaker correct many odd phrases. There are repeated instances such as "died for" instead of "died from", "besides" instead of "in addition", "found differently" instead of "found to be different," and most annoying, "high risk of OS and CSS". The patients are not at high risk of overall survival, but at high risk of lower overall survival (more likely to die) and cancer specific survival.

Response: Thank you for your valuable comments. We have checked and revised the grammatical mistakes in the manuscript, and invited a native English speaker to help us. We hope the revised manuscript could meet your standards.

- In the Methods section, the authors state the database used from SEER was from 2000-2019, but in Figure 1, it is 2010-2019.

Response: Thank you for your comments. We are very sorry for the clerical error, and have revised the time of data collection we used, that the correct time was 2010-2019.

There is confusion as to what HER2 means; in Variables collection it states "...and hormonal estrogen receptor (HER)," and this is repeated in the Discussion. HER stands for Human Epidural Growth Factor receptor, not hormone estrogen receptor; this is an egregious error.

Response: Thank you for your comments. We are very sorry for this egregious error, and have revised the “hormonal estrogen receptor” into “human epidural growth factor receptor” in the manuscript.
